# Enhanced Extraction of Blood and Tissue Time-Activity Curves in Cardiac Mouse FDG PET Imaging by Means of Constrained Nonnegative Matrix Factorization

**DOI:** 10.1155/2023/5366733

**Published:** 2023-06-15

**Authors:** Otman Sarrhini, Pedro D'Orléans-Juste, Jacques A. Rousseau, Jean-François Beaudoin, Roger Lecomte

**Affiliations:** ^1^Sherbrooke Molecular Imaging Center, Research Center of the Sherbrooke University Hospital (CRCHUS), Sherbrooke, QC, Canada; ^2^Department of Pharmacology-Physiology, Faculty of Medicine and Health Sciences, Université de Sherbrooke, Sherbrooke, QC, Canada; ^3^Department of Nuclear Medicine and Radiobiology, Faculty of Medicine and Health Sciences, Université de Sherbrooke, Sherbrooke, QC, Canada

## Abstract

We propose an enhanced method to accurately retrieve time-activity curves (TACs) of blood and tissue from dynamic 2-deoxy-2-[^18^F]fluoro-D-glucose ([^18^F]FDG) positron emission tomography (PET) cardiac images of mice. The method is noninvasive and consists of using a constrained nonnegative matrix factorization algorithm (CNMF) applied to the matrix (*A*) containing the intensity values of the voxels of the left ventricle (LV) PET image. CNMF factorizes *A* into nonnegative matrices *H* and *W*, respectively, representing the physiological factors (blood and tissue) and their associated weights, by minimizing an extended cost function. We verified our method on 32 C57BL/6 mice, 14 of them with acute myocardial infarction (AMI). With CNMF, we could break down the mouse LV into myocardial and blood pool images. Their corresponding TACs were used in kinetic modeling to readily determine the [^18^F]FDG influx constant (*K*_*i*_) required to compute the myocardial metabolic rate of glucose. The calculated *K*_*i*_ values using CNMF for the heart of control mice were in good agreement with those published in the literature. Significant differences in *K*_*i*_ values for the heart of control and AMI mice were found using CNMF. The values of the elements of *W* agreed well with the LV structural changes induced by ligation of the left coronary artery. CNMF was compared with the recently published method based on robust unmixing of dynamic sequences using regions of interest (RUDUR). A clear improvement of signal separation was observed with CNMF compared to the RUDUR method.

## 1. Introduction

It is well established that positron emission tomography (PET) is a powerful imaging modality that measures the local concentration of radiotracers. Thus, given a dynamic sequence of PET images, one can visualize and precisely quantify the involved metabolic processes following the injection of radiolabeled compounds.

The mouse is increasingly being used as the preferred animal model for many in vivo molecular imaging studies [1, 2]. The use of well-validated tracer kinetic models with dynamic PET sequences allows estimating physiological parameters [3]; however, suitable corrections are required to obtain the accurate blood and tissue time-activity curves (TACs) needed in kinetic modeling, especially in mouse studies. In fact, despite the improvement of the spatial resolution of modern scanners (~1-1.5 mm [4]), the partial volume effect (PVE) remains a major impediment for imaging small structures [5, 6].

In dynamic cardiac PET studies, the input function and tissue TACs, describing the arterial plasma and myocardial wall radioactivity concentrations, are necessary for myocardial blood flow and metabolic rate measurements. However, in the mouse model, due to the small size of the heart, the measured TACs will inevitably lead to inaccurate estimation of rate parameters if appropriate corrections are not applied.

Manual blood sampling, considered as the gold standard, is laborious and inherently unable to properly capture the initial peak of the input function, especially during a rapid bolus injection. Furthermore, only up to 10% (e.g., ~0.2 mL) of the total blood volume can be taken from a mouse without significantly altering its physiologic conditions [7], thus limiting longitudinal studies. Automated blood sampling methods have been developed to determine the input function [8, 9] with a potential for smaller blood volumes. However, the input function from these methods needs to be corrected for propagation delay and dispersion [10]. A hybrid image and blood sampling method was developed to derive input function for quantification of microPET mouse data [11]. This method used the recovered left ventricle (LV) TAC peak linked with 5-6 optimally placed blood sample points. Another hybrid image-derived input function was proposed using the LV peak followed by the remainder of the liver TAC normalized to a 60 min arterial blood sample [12]. Thorn et al. [13] have reported a methodology for measuring [^18^F]FDG myocardial glucose uptake using vena cava image-derived input function corrected for PVE. They used contrast computed tomography (CT) and modeling to determine the vena cava recovery coefficient (RC). Two machine learning-derived input function models have been recently proposed by Kuttner et al. [14]. These models, when properly trained, can predict the real input function. Despite their validation, none of these methods address the myocardial TAC correction, which is also affected by the PVE.

Fang and Muzic [15] proposed a simultaneous estimation model to correct both the LV cavity (LVC) and the myocardial TACs for PVE in small animal PET studies. Their method assumes that the measured LV and myocardial TACs can be expressed as a weighted sum of a modeled blood and tissue activity. This model, while satisfactorily describing the spillover issues, tries to estimate 15 parameters simultaneously by minimizing a constrained objective function and, as such, may lead to uncertainty in parameter estimation. Li and Kundu have developed a hybrid optimization method based on the artificial immune system algorithm to reduce the uncertainty caused by guess values in simultaneous estimation models [16]. Locke et al. [17] applied the ordered subset expectation maximization—maximum a posteriori algorithm and “froze” the heart around the diastolic phase to reduce the cross-contamination between LVC and myocardium and then boosted the obtained TACs by a predetermined RC. Their reconstruction, however, takes several hours per dynamic image, which makes the approach cumbersome for high-throughput cardiac research.

Various techniques have been investigated to separate different physiological signals in PET images. These techniques comprise independent component analysis (ICA) [18], principal component analysis (PCA) [19], and factor analysis of dynamic sequences (FADS) [20, 21]. These approaches assume that, due to the PVE, the physiological signals are mixed up in each voxel of the image and therefore can be extracted separately in a mathematical framework. Several versions have been developed in which, in addition to the positivity constraint [20, 22], improvements have been made to better separate the factors and thus reduce the ambiguity on the solution, either with spatial regularization [23], or by minimizing the overlap between the factor images [24, 25] or else by incorporating a priori physiological knowledge [26, 27]. These algorithms, however, need to be validated in a high cross-contamination context such as in cardiac mouse PET imaging.

Nonnegative matrix factorization (NMF) is another technique to separate overlapped signals in dynamic PET images. NMF was originally developed by Lee and Seung in their Nature article [28] to learn parts of faces and semantic features of text. They implemented update rules to minimize the Euclidian distance and the Kullback-Leibler divergence [29]. Various alternative minimization strategies have been suggested to use with NMF [30, 31].

Lee et al. [32] reported in a preliminary study that NMF would be feasible for image segmentation and factor extraction from dynamic PET image sequences in nuclear medicine. Subsequently, several NMF-based approaches have been suggested to extract the input curve from dynamic PET images of mice [33–35].

Recently, robust unmixing of dynamic sequences using regions of interest (RUDUR) algorithm was proposed in which knowledge of regions of interest (ROIs) was integrated as soft constraints in the objective function to overcome nonuniqueness issues [36]. Compared to other competitive methods [20, 24, 37, 38], RUDUR showed a significant improvement of the source separation.

In this study, we present a method that uses a constrained NMF algorithm incorporating normalization and regularization to properly extract LVC and myocardium TACs from [^18^F]FDG PET cardiac mouse images. These TACs, corrected for PVE and cross-contamination without extra steps, were used to assess the myocardial [^18^F]FDG influx constant *K*_*i*_ in normal and infarcted mice. The proposed CNMF method was compared qualitatively on the retrieved TACs and quantitatively on the calculated *K*_*i*_ constants to the RUDUR method [36] as a validation in the absence of reliable blood sampled input function.

## 2. Theoretical Background

### 2.1. Constrained Nonnegative Matrix Factorization

As a matrix factorization method, NMF can be stated in general form as follows: given a nonnegative matrix *A* (*n* × *m*) and a positive integer *r* < <min (*n*, *m*), find iteratively nonnegative matrices *W* (*n* × *r*) and *H* (*r* × *m*) such that *A* ≈ *WH* by minimizing an objective function *f*(*W*, *H*). In PET imaging, the rows of *H* represent the physiological factors and the columns of *W* contain their corresponding weights in each voxel. *n* is the number of voxels included in *A*, *r* is the number of factors, and *m* is the number of time-frames. Because of inevitable statistical uncertainties in the PET image, and in order to enforce desired smoothness in the computed solution, we used the constrained NMF (CNMF) of Pauca et al. [39]. Briefly, CNMF tries to find two positive matrices *W* and *H* by minimizing the objective function:

(1) $$\begin{array}{c} f\left(W,H\right)=\frac{1}{2}\left({\left\|A-WH\right\|}_{F}^{2}+\alpha {\left\|W\right\|}_{F}^{2}+\beta {\left\|H\right\|}_{F}^{2}\right). \end{array}$$

*A* is the *n* × *m* matrix with elements *A*_*ij*_ = *P*_*ij*_/∑_*l*=1_^*m*^*P*_*il*_, where *P*_*ij*_ is the *ij*^th^ element of the matrix *P*(*n* × *m*) of PET data from the whole LV ROI (including myocardium and LVC), *α* and *β* are the positive regularization parameters that balance the trade-off between the approximation error and the constraints, and ${\left\|X\right\|}_{F}=\sqrt{\sum_{i=1}^{n}\sum_{j=1}^{m}x_{ij}^{2}}$ is the Frobenius norm [40] of *X*.

The minimization of (1) is performed by alternating nonnegative least squares solution using the block principal pivoting algorithm of Kim and Park [41] applied to iteratively solve

(2a) $$\begin{array}{c} \underset{H\geq 0}{\min}{\left\|\left(\begin{array}{c} W\\ \sqrt{\beta }I_{r} \end{array}\right)H-\left(\begin{array}{c} A\\ 0_{r\times m} \end{array}\right)\right\|}_{F}^{2}, \end{array}$$

with fixed *W*, and

(2b) $$\begin{array}{c} \underset{W\geq 0}{\min}{\left\|\left(\begin{array}{c} H^{t}\\ \sqrt{\alpha }I_{r} \end{array}\right)W^{\text{t}}-\left(\begin{array}{c} A^{t}\\ 0_{r\times n} \end{array}\right)\right\|}_{F}^{2}, \end{array}$$

with fixed *H*. *I*_*r*_ is an *r* × *r* identity matrix, and 0_*r*×*m*_ and 0_*r*×*n*_ are the zero matrices of size *r* × *m* and *r* × *n*. *X*^*t*^ is the transpose matrix of *X*.

Normally, the mixed signals in *A* are originating mainly from the myocardium, the left and right ventricle cavities. However, the right ventricle to left ventricle transit time in ~30 g mice was estimated to 1.01 ± 0.3 seconds [42]. Due to the used frame sequence (≥5 s), we believe that only 2 factors need to be considered in this study, namely, the tissue factor from the myocardium and the blood factor from the LVC (i.e., *r* = 2).

As for any iterative algorithm, *H* and *W* must be initialized. For this, we defined a *n* × *r* matrix *M* which is a binary mask representing the myocardial and LVC ROIs as follows [36]:

(3) $$\begin{array}{c} M_{ik}=\left\{\begin{array}{cc} 1 & \text{if the }i^{\text{th}}\text{ voxel is in the }k^{\text{th}}\text{ ROI},\\ 0 & \text{otherwise}. \end{array}\right. \end{array}$$

**Figure 1:**
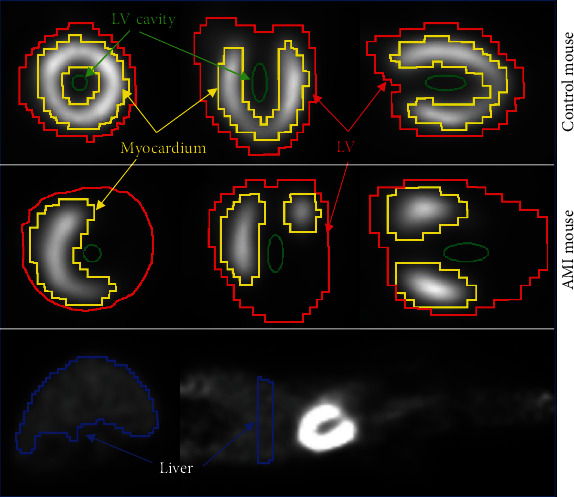
[^18^F]FDG images of control (first row) and AMI (second row) C57BL/6 mice showing typical ROIs used in cardiac [^18^F]FDG PET image analysis. The liver ROI is presented in the third row on the transaxial (left) and coronal (right) views. The ROI names are given in the legend.

*H* and *W* are then initialized as follows:

(4a) $$\begin{array}{c} H_{kj}^{\left(0\right)}=\frac{\sum_{i}A_{ij}}{\sum_{i}M_{ik}},\text{such that }M_{ik}=1, \end{array}$$

(4b) $$\begin{array}{c} W_{ik}^{\left(0\right)}=\frac{1}{1+\underset{j}{\min}{\text{dist}}_{\text{Euc}}\left(i,j\right)},\text{such that }M_{jk}=1, \end{array}$$

(4c) $$\begin{array}{c} \overset{r}{\underset{k=1}{\sum }}W_{ik}^{\left(0\right)}=1,\forall 1\leq i\leq n, \end{array}$$

where dist_Euc_(*i*, *j*) is the Euclidean distance between the *i*^th^ and *j*^th^ voxels. In other words, *H* is initialized with the normalized TACs obtained from crude ROIs drawn on myocardium and LVC ROIs (Figure 1), while the Euclidian distance between voxels and ROIs is considered for *W* initialization. Specifically, *W*_*ik*_^(0)^ = 1 if and only if the *i*^th^ voxel is in the *k*^th^ ROI; otherwise, the greater the distance between the *i*^th^ voxel and the *k*^th^ ROI, the smaller *W*_*ik*_^(0)^ is. This initialization ensures reasonable starting weights for the voxels according to their distance to a region of interest.

Since the iterative algorithm of Equation (2a) does not necessarily maintain the relationship of Equation (4c), we applied the scaling procedure described in [43]. Briefly, a vector *v* was calculated to rescale *W* and *H*. The *j*^th^ element (*j* = 1 ⋯ *r*) of the vector *v* is given by

(5) $$\begin{array}{c} v_{j}={\left(W^{\text{t}}W\right)}^{-1}{\left(\underset{i}{\sum }W_{ij}\right)}^{t}. \end{array}$$

In other words, the transposed vector *v*^*t*^ is elementwise multiplied with each row of *W*, and the inverse *v*^−1^ is elementwise multiplied with each column of *H* so that the product *WH* will not change. This scaling step is useful when applying the NMF algorithm to PET images so that each row of *W* will sum to 1 (Equation (4c)), thus ensuring that the various signals from different tissues coexist in each voxel but with different weights. The matrix *H* can be regarded as the physiological factors and *W*, as their corresponding weights in each voxel.

Our objective is to extract the true activity of the factors through CNMF. Owing to the normalized units of the obtained factors, the true magnitude of the CNMF TAC of the *k*^th^ factor at time *j* was determined by

(6) $$\begin{array}{c} {\text{CNMFTAC}}_{k}\left(j\right)=H_{kj}\frac{\sum_{i}\sum_{l=1}^{m}P_{il}}{\sum_{i}M_{ik}},\text{such that }M_{ik}=1, \end{array}$$

with *P* representing the *n* × *m* matrix of measured PET data from the LV ROI.

Since *α* and *β* are the parameters that control smoothness and sparsity of *W* and *H*, respectively [41], they were chosen such that the tail of the calculated CNMF blood factor of Equation (6) would match the activity value from the blood sample. Thus, Equation (6) returned CNMF blood (*C*_*B*_) and tissue (*C*_*T*_) TACs corrected for PVE and cross-contamination. The unconstrained NMF (UNMF) data can be extracted by setting *α* = 0 and *β* = 0.

For the RUDUR algorithm [36], the same starting point *W*^(0)^ and *H*^(0)^ was used together with the same input matrix *A*. Equation (6) was also applied to the RUDUR results to obtain RUDUR blood and tissue TACs. We used MATLAB programming language for algorithm implementation. The source code is available at https://github.com/osarrhini/CNMF.

### 2.2. [^18^F]FDG Three-Compartment Kinetic Model

The three-compartment model (3CM) is commonly used to assess the [^18^F]FDG metabolism [44]. The mathematical representative equations of this model are

(7a) $$\begin{array}{c} \frac{dC_{f}\left(t\right)}{dt}=K_{1}C_{p}\left(t\right)-\left(k_{2}+k_{3}\right)C_{f}\left(t\right)+k_{4}C_{m}\left(t\right), \end{array}$$

(7b) $$\begin{array}{c} \frac{dC_{m}\left(t\right)}{dt}=k_{3}C_{f}\left(t\right)-k_{4}C_{m}\left(t\right), \end{array}$$

(7c) $$\begin{array}{c} C_{T}\left(t\right)=\left(1-v_{B}\right)\left(C_{f}\left(t\right)+C_{m}\left(t\right)\right)+v_{B}C_{B}\left(t\right), \end{array}$$

where *C*_*p*_(*t*), *C*_*f*_(*t*), and *C*_*m*_(*t*) are, respectively, the [^18^F]FDG concentration in plasma, the free [^18^F]FDG concentration in tissue, and the concentration of the metabolized [^18^F]FDG-6-phosphate in tissue. *C*_*B*_(*t*) and *C*_*T*_(*t*) are either the CNMF or RUDUR blood and tissue TACs. *v*_*B*_ (mL/g) is the tissue blood volume. *K*_1_ (mL/g/min) and *k*_2_ (min^−1^) are the rate constants characterizing the [^18^F]FDG forward and reverse capillary membrane transport between plasma and tissue; *k*_3_ (min^−1^) and *k*_4_ (min^−1^) are the rate constants depicting the phosphorylation of [^18^F]FDG and the dephosphorylation of [^18^F]FDG-6-phosphate. The plasma [^18^F]FDG activity *C*_*p*_(*t*) was determined from *C*_*B*_(*t*) by applying blood-to-plasma activity conversion as described in [8]

(8) $$\begin{array}{c} C_{p}\left(t\right)=C_{B}\left(t\right)\left(0.386e^{-0.19t}+1.165\right). \end{array}$$

Once the parameters (*K*_1_, *k*_2_, *k*_3_, *k*_4_, and *v*_*B*_) are estimated by least squares fitting using PMOD's dedicated tools (Version 3.9, PMOD Technologies, Switzerland), we calculated the [^18^F]FDG influx constant *K*_*i*_ = *K*_1_*k*_3_/(*k*_2_ + *k*_3_).

## 3. Materials and Methods

### 3.1. Animal Model

Thirty-two C57BL/6 mice (30.4 ± 2.4 g), on a Teklad-irradiated global soy, protein-free extruded diet (Inotiv, catalog no T2920X.10), were divided into two groups: healthy control mice (CTRL) (*n* = 18) and mice with acute myocardial infarction (AMI) (*n* = 14) induced by surgical occlusion of the left coronary artery [45]. The AMI group was itself divided into two subgroups: mice scanned for 3 days (AMI3d) (*n* = 8) and mice scanned for 14 days (AMI14d) (*n* = 6) after AMI induction. During PET measurements, the mice were maintained under mild anesthesia (~1.0-1.5% isoflurane and 1.0-1.5 L/min oxygen), and their body temperature was controlled with a heating pad. All mouse experiments were conducted in accordance with the recommendations of the Canadian Council on Animal Care and with the approval of the University of Sherbrooke Ethics Committee for Animal Experiments (Protocol No. 199-13R).

### 3.2. PET Measurements

PET data were acquired in list-mode using the Triumph™ dual modality platform (Gamma Medica, Inc., Northridge, CA), consisting of a LabPET8 avalanche photodiode-based digital PET scanner with 7.5 cm axial and 10 cm transaxial field of view and a high-resolution X-ray CT scanner. The spatial resolution measured at 5 mm from the center of the PET scanner was 1.51 mm and 1.62 mm in the radial and tangential direction, respectively, with 2D-filtered back projection reconstructed images. Since the LabPET8 scanner design is optimized for iterative reconstruction algorithm, hot spots down to 1 mm can be resolved in iteratively reconstructed 3D images [46].

Each mouse was positioned on the scanner bed, head first supine, making sure that the heart is approximately at the center of the scanner field of view. The 45-minute list-mode acquisitions were started 30 s before administration in the tail vein of 7.9 ± 1.8 MBq in 100 *μ*L of [^18^F]FDG at a rate of 200 *μ*L/min using an automatic pump. One blood sample of 15.6 ± 7.4 *μ*L was manually collected in a preweighted tube from the caudal artery 9.3 ± 3.2 min after the end of the acquisition. Radioactivity in the blood sample was measured in a gamma counter, which was cross-calibrated with the scanner, and used as a reference for the CNMF and RUDUR image-derived blood TACs. Images were reconstructed on a 120 × 120 × 128 matrix with a 0.5 mm × 0.5 mm × 0.6 mm voxel size using 20 iterations of the 3D maximum likelihood expectation maximization algorithm implementing a 3D model of the physical detector response [47]. Frame durations for the reconstructed images were 16 × 5 s, 7 × 10 s, 8 × 30 s, 1 × 60 s, 5 × 150 s, and 5 × 300 s. All PET images were corrected for ^18^F physical radionuclide decay, dead time, and variations in crystal detection efficiency. A calibration factor was determined using a 3 cm diameter cylindrical phantom loaded with known activity and was used to convert image counts/s/voxel to percent of injected dose per gram (%ID/g) assuming a tissue density of 1 g/cm^3^.

### 3.3. Regions of Interest Delineation

The reconstructed images were reoriented to short axis using the PMOD cardiac PET tool. The last 5 frames of the reoriented images were averaged and used to draw, by thresholding, a region of interest around the mouse heart using PMOD. The threshold was visually set so that only the whole LV was included in the ROI. The LV ROI was then applied to the dynamic reoriented image series, and the obtained intensity of voxels was reorganized into a matrix (*n* × *m*), which in turn was normalized to obtain the *n* × *m* matrix *A* with *n* voxels and *m* frames. Each row of *A* sums to 1 and contains the normalized activity of a voxel as a function of time. This matrix was used as the input of the CNMF algorithm. Two other ROIs were drawn over the myocardial wall and the LVC. For the latter ROI, an ellipse of 1.3 mm × 1.3 mm × 3 mm was manually centered in the LVC while an interactive thresholding was used to delineate appropriate hot myocardial contours. These two ROIs are used to estimate spillover and RC values in the myocardium and the LVC. Before image reorientation, a liver ROI was also drawn by thresholding the transaxial plane crossing approximately the middle of the liver. Figure 1 shows typical ROIs used in this study.

### 3.4. Comparison of CNMF and RUDUR

The CNMF and RUDUR TACs of Equation (6) were compared for each mouse using the root mean square error (RMSE) defined as

(9) $$\begin{array}{c} {\text{RMSE}}_{k}=\sqrt{\frac{\sum_{t=1}^{m}{\left({\text{CNMFTAC}}_{k}\left(t\right)-{\text{RUDURTAC}}_{k}\left(t\right)\right)}^{2}}{m}}, \end{array}$$

where *k* denotes blood or tissue TAC. We also compared, for each group of mice, the mean values of CNMF and RUDUR weights (elements of matrix *W*) in LVC and in the myocardium. The relationship between the CNMF and RUDUR *K*_*i*_ was assessed through the linear regression and Bland-Altman plots.

## 4. Statistical Analysis

All values are reported as mean ± standard deviation. Differences in *K*_*i*_ values between CNMF and RUDUR were evaluated for statistical significance using paired Student's *t*-test. The mean values of weights (*W* matrix) in myocardium and LVC ROIs were compared using one-way ANOVA with a Tukey's multiple comparison test. To highlight the effect of the AMI on the myocardial metabolism of glucose, we used one-way ANOVA with a Tukey's multiple comparison test to compare the *K*_*i*_ values between CTRL, AMI3d, and AMI14d groups obtained either with CNMF or RUDUR algorithms. Equal variances were assumed for all analyses. A *p* value of less than 0.05 was considered statistically significant. All statistical analyses were performed using GraphPad Prism version 8.3.1 (GraphPad Software, San Diego CA, http://www.graphpad.com).

## 5. Results

**Figure 2:**
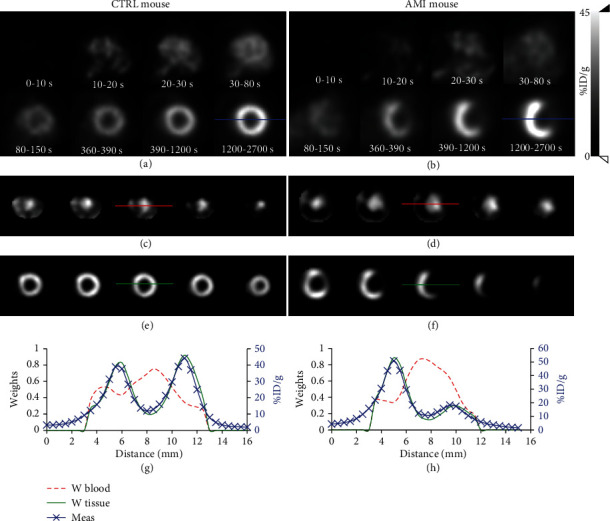
Typical short axis slice of [^18^F]FDG dynamic PET images of a CTRL mouse heart (a) and a mouse with AMI (b) showing the progression of the radiotracer from the vasculature (0-20 s) to the cardiac ventricular chambers (20-30 s) through the lungs (30-80 s) and finally its accumulation in the myocardium. The last frame (1200-2700 s) shows clearly that the [^18^F]FDG cardiac uptake is uniform in the CTRL mouse, while the AMI mouse shows an infarcted area of absent uptake. (c–h) depict an example of CNMF application to a CTRL mouse (c, e, g) and AMI mouse (d, f, h). Typical slices of basis images (*W* matrix) are shown for blood (c, d) and tissue factors (e, f), taken approximately every 1 mm from the basal plane (left) towards the apex (right). (g, h) display the profiles across the matrix *W* and the last frame (1200-2700 s) at the positions indicated by the corresponding colored lines. (g, h) The left *y*-axis is for *W* profiles while the right *y*-axis is for the measured images profile.

Figures 2(a) and 2(b) show an example of dynamic PET images of a CTRL and an AMI mouse LV. We note the increased [^18^F]FDG uptake in the myocardium as a function of time. However, this uptake is markedly reduced for the infarcted segment of the myocardium (Figure 2(b)). *W* and *H* were obtained for the images of all 32 mice. *W* was reshaped into the original image grid to show the distribution of weights. Thus, blood and tissue components can be easily distinguished as illustrated in Figures 2(c) and 2(e) for a CTRL mouse and in Figures 2(d) and 2(f) for an AMI mouse. As can be seen from the profiles across the *W* matrix for the blood component in Figures 2(c) and 2(d) and the tissue component in Figures 2(e) and 2(f), the CNMF algorithm works well in isolating the two components even for the AMI mouse (see Figures 2(g) and 2(h)).

**Figure 3:**
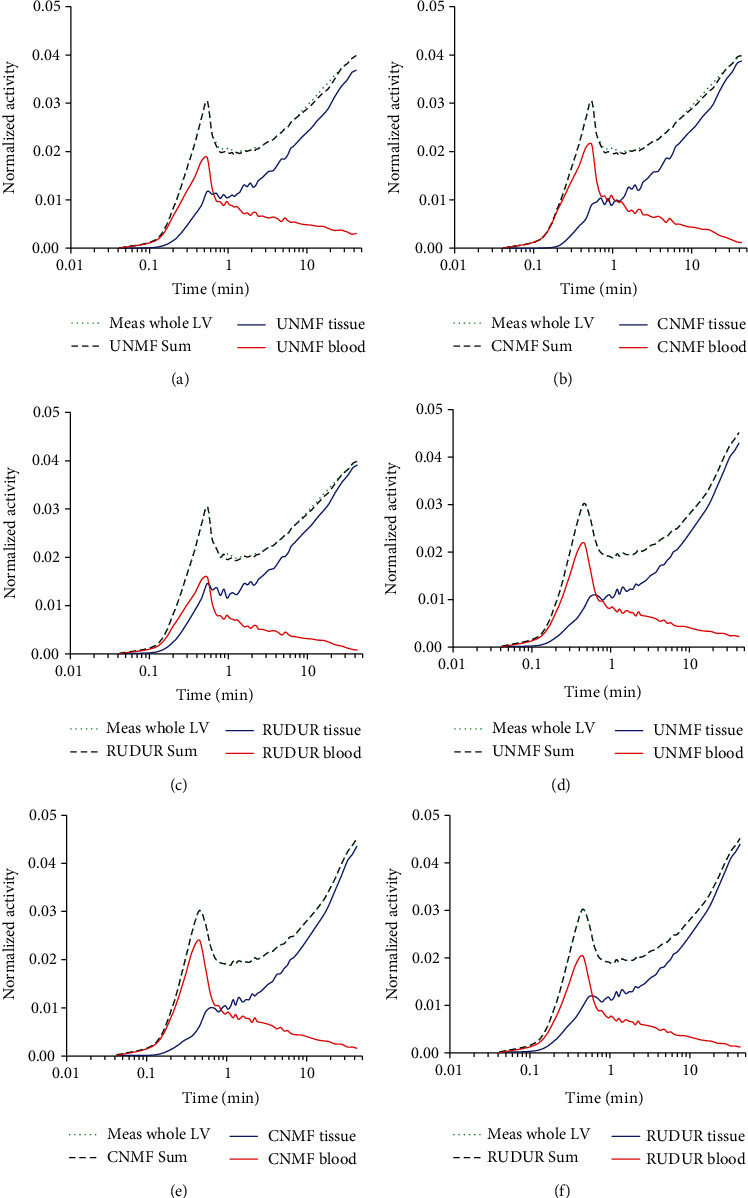
The mean of *WH* product over the whole LV voxels is calculated and displayed as a function of time for a CTRL (a, b, c) and an AMI3d mouse (d, e, f) using UNMF (a, d), CNMF (b, e), and RUDUR (c, f). The mixed signals in PET images (Meas whole LV) are separated into blood (UNMF blood, CNMF blood, and RUDUR blood) and tissue (UNMF tissue, CNMF tissue, and RUDUR tissue) factors. As can be seen, the sum of the factors (either from UNMF, CNMF, or RUDUR) agrees well with the measured data. The semilog chart is used to better display the difference between the various curves.

Figure 3 shows the plot of the *WH* product, as a function of time, for each factor (blood and tissue) and averaged over the whole LV voxels. Blood and tissue factors, from UNMF (first column), CNMF (second column), and RUDUR (third column), for a CTRL (upper row) and an AMI mouse (bottom row), are clearly separated and show the expected trends. Moreover, the sum of the two factors agrees well with the mean of the measured activity over the whole LV for all three methods. There is, however, a clear unmixing improvement when using CNMF, especially for the CTRL mouse (Figure 3(b)), but to a less extent for the AMI mouse due to myocardial hypertrophy.

**Figure 4:**
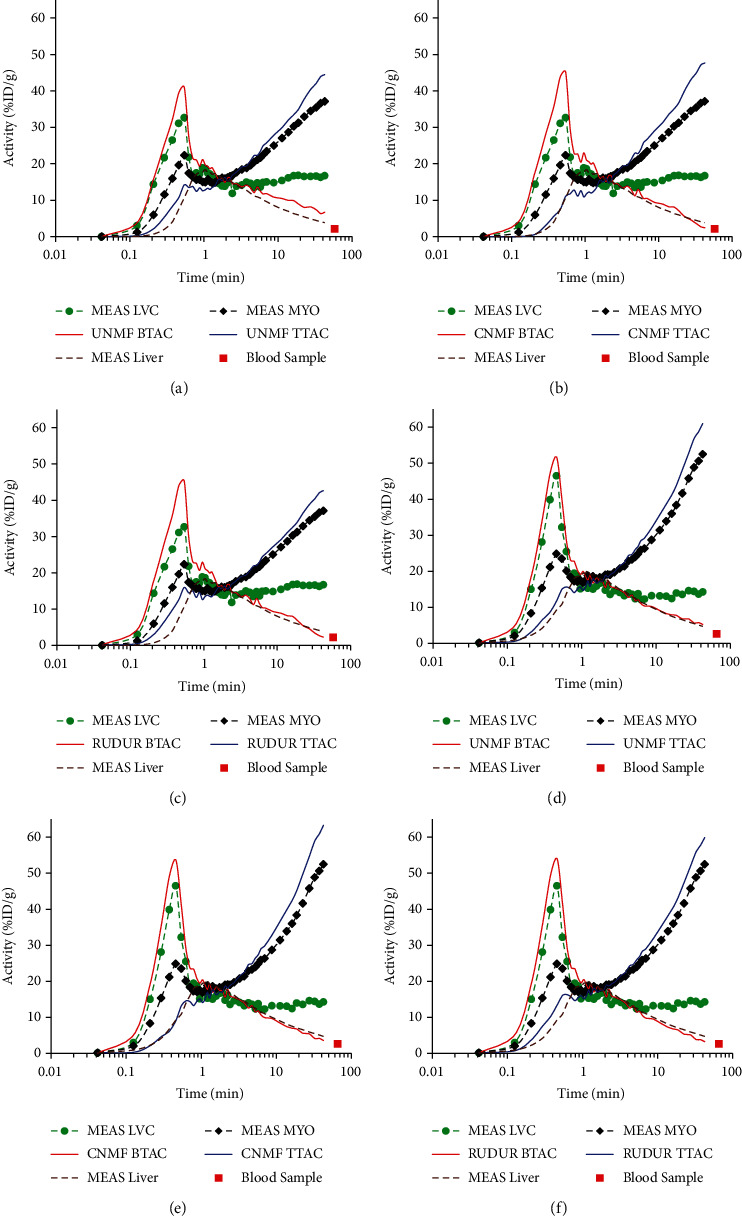
Time-activity curves obtained from dynamic images of a CTRL (a, b, c) and an AMI3d mouse (d, e, f). The measured TACs (MEAS LVC, MEAS MYO, and MEAS Liver) are obtained by means of ROIs drawn on the respective organs while UNMF (a, d), CNMF (b, e), and RUDUR (c, f) TACs were obtained with their respective methods. The last blood sample (blood sample) is used as a reference to adjust the CNMF solution via the *α* and *β* parameters, while the RUDUR solution is adjusted via its own regularization parameters [36]. The tail of the liver TAC can also be used instead of the blood sample. The semilog chart is used to better display the difference between the various curves. The same figure in which the curves were zoomed around the one-minute time points is provided in Supplemental Data to better show the difference between the different curves.

**Figure 5:**
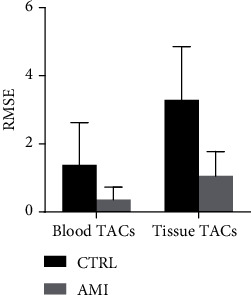
Mean with SD bar plot of RMSE between CNMF and RUDUR TACs as determined from Equation (9). Blood TACs showed less difference between CNMF and RUDUR compared to tissue TACs. Better agreement between the two methods is observed in the AMI groups.

Typical blood (BTAC) and tissue (TTAC) time-activity curves calculated using the three same methods are shown in Figure 4 together with the measured TACs for a CTRL (upper row) and an AMI mouse (bottom row). When visually comparing the measured tissue (MEAS MYO) and blood (MEAS LVC) to their respective calculated counterparts, we can see that all three methods reduce cross-contamination by recovering the signals in the early BTACs and late TTACs. However, the corrected CNMF TTACs show significantly reduced initial peaks due to the contamination from the LVC, while the corrected CNMF BTACs drop normally after 2 to 3 min postinjection, closely following the measured liver TAC and nearly intercepting the blood sample, unlike the UNMF BTACs (Figures 4(a) and 4(d)). For this reason, the measured liver TAC could be considered as an appropriate approximation for the CNMF solution at time beyond ~5-10 min and a safe reference beyond ~20-40 min postinjection where [^18^F]FDG retention in the liver can be considered negligible. The RUDUR method performs almost the same as CNMF approach, but with a slightly larger early peak in the RUDUR TTAC and a slightly poorer agreement of the extrapolated RUDUR BTAC with the late blood sample. The difference between CNMF and RUDUR TACs is reported in Figure 5 in terms of RMSE (Equation (9)).

**Figure 6:**
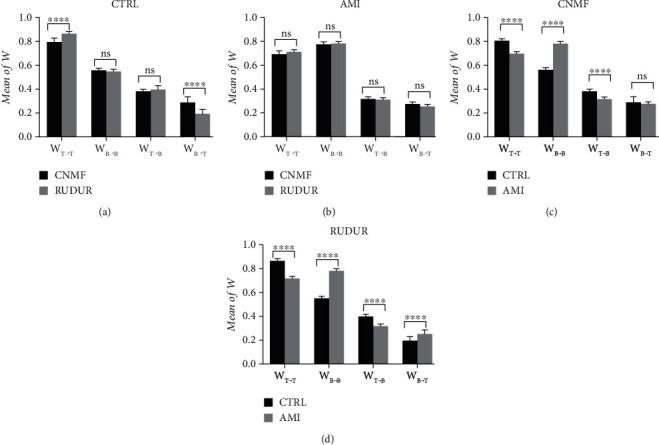
Mean value of CNMF and RUDUR weights (*W* matrix) as determined for CTRL (a) and AMI mouse hearts (b). The difference between the CTRL and AMI weights is displayed in (c) for CNMF and (d) for RUDUR. *W*_*i*⟶*j*_ is the mean value of the *i*^th^ basis image over the *j*^th^ ROI (*i*, *j* = *T*, *B*). *W*_*T*⟶*T*_ and *W*_*B*⟶*B*_ can be assimilated to the myocardial and LVC RCs, respectively, while *W*_*T*⟶*B*_ and *W*_*B*⟶*T*_ represent the spill-in fraction from the myocardium to LVC and the spill-out fraction from the LVC to the myocardium, respectively. In this figure, we have pooled together the AMI3d and AMI14d groups. ^∗∗∗∗^ denotes *p* < 0.0001.

Since the weights in *W* can be assimilated either to the RCs or to the spillover fractions at the voxel scale, we calculated the mean of *W* over the myocardium and LVC for each CNMF and RUDUR factor. The results of this calculation are summarized in Figure 6. The AMI3d and AMI14d subgroups were merged for this analysis since these subjects should have experienced similar structural changes after surgery. Thus, *W*_*T*⟶*T*_ and *W*_*B*⟶*B*_ are, respectively, the mean of the tissue factor weights over the myocardium and the mean of the blood factor weights over the LVC. They represent the RC in the myocardium and in the LVC. In the same way, *W*_*T*⟶*B*_ and *W*_*B*⟶*T*_ are, respectively, the mean of the tissue factor weights in the LVC and the mean of the blood factor weights in the myocardium. They represent the spillover fraction from myocardium to LVC and vice versa. There is a fairly good agreement for most of the weights between CNMF and RUDUR, with the noteworthy exception of a significantly lower *W*_*B*⟶*T*_ concomitant with a higher *W*_*T*⟶*T*_ in the CTRL mice with the latter method. *W*_*T*⟶*T*_ is larger for CTRL than for the AMI group (0.799 ± 0.027 vs. 0.698 ± 0.023 with CNMF and 0.868 ± 0.015 vs. 0.716 ± 0.021 with RUDUR) while *W*_*B*⟶*B*_ shows the opposite trend (0.562 ± 0.016 vs. 0.779 ± 0.022 with CNMF and 0.552 ± 0.017 vs. 0.782 ± 0.021 with RUDUR) (*p* < 0.0001). These results are in a good agreement with the observed structural changes, namely, myocardial wall thinning and LVC dilatation, following myocardial infarction induction in mice [48]. For the spillover fractions, *W*_*T*⟶*B*_ shows a significant difference (*p* < 0.0001) between the CTRL (0.385 ± 0.019 with CNMF and 0.402 ± 0.027 with RUDUR) and AMI groups (0.319 ± 0.019 with CNMF and 0.315 ± 0.023 with RUDUR). No significant difference was seen between the CTRL and AMI groups for *W*_*B*⟶*T*_ with CNMF (0.295 ± 0.042 vs. 0.276 ± 0.026; *p* = 0.3672), whereas, with RUDUR, *W*_*B*⟶*T*_ displayed a significant difference (*p* < 0.0001) between the CTRL and AMI groups (0.195 ± 0.037 vs. 0.257 ± 0.024). All comparisons of mean values of *W* are summarized in Tables 1 and 2.

**Figure 7:**
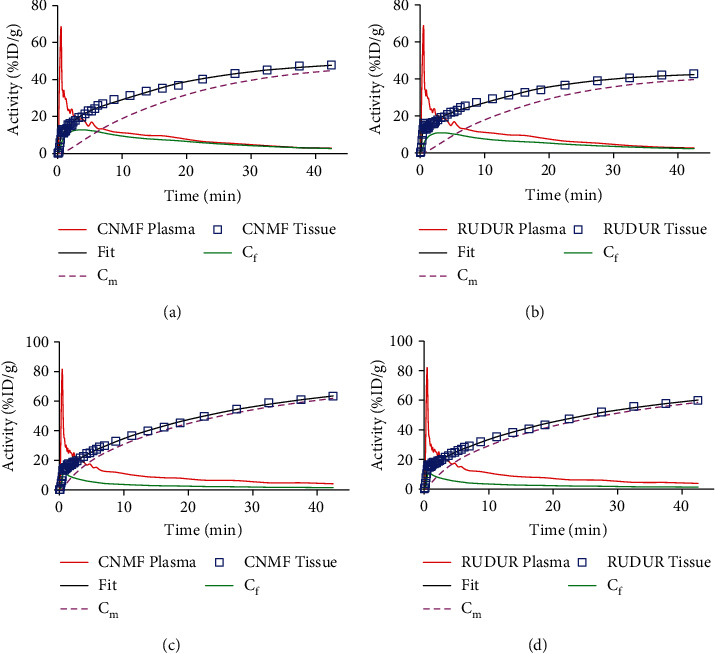
3CM curve fitting plot examples for a CTRL (a, b) and for an AMI3d mouse (c, d) using CNMF (a, c) and RUDUR TACs (b, d). Both methods show comparable fitting results.

**Figure 8:**
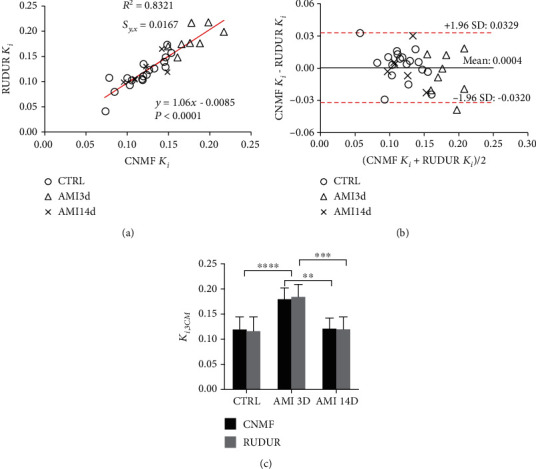
Correlation between [^18^F]FDG influx constant *K*_*i*_ determined from the 3CM analysis using CNMF and RUDUR: linear regression (a) and Bland-Altman plot (b). Both methods show significant difference in *K*_*i*_ values between CTRL and AMI3d groups (*p* < 0.0001) and between AMI3d and AMI14d (*p* < 0.005 for CNMF and *p* < 0.0005 for RUDUR), but no difference was seen between CTRL and AMI14d groups as shown in (c).

Typical 3CM curve fitting plots are shown in Figure 7 for CNMF (left column) and RUDUR blood and tissue TACs (right column). The calculated [^18^F]FDG influx constant *K*_*i*_ for CTRL and AMI mice at 3 days and 14 days postinfarction is summarized in Figure 8. Paired Student's *t*-test revealed no significant difference between the CNMF and RUDUR calculated *K*_*i*_ values. This is further illustrated with the linear regression (Figure 8(a)) and Bland-Altman (Figure 8(b)) plots. As can be seen in Figure 8(c), *K*_*i*_ values are found to be significantly higher in AMI3d mice under acute myocardial infarction, compared to CTRL mice (*p* < 0.0001), whereas the AMI14d mice that progressed to a chronic condition show approximately the same values of *K*_*i*_ as the CTRL mice. Detailed results are reported in Supplemental Data (available here).

## 6. Discussion

In cardiac mouse PET imaging, TACs can be obtained by drawing ROIs on the LVC and on the myocardium. However, due to the small size of the mouse heart, PVE and cross-contamination between these two adjacent regions are expected. Accordingly, the signals from LVC and myocardium coexist in each voxel of the heart PET images, especially for tracers that show physiological uptake in the myocardium, such as [^18^F]FDG [49], ^13^NH_3_ [50], and ^11^C-acetate [51]. Thus, adequate correction of PVE and cross-contamination in TACs is a requirement before using kinetic models [52, 53].

In the present work, we explored the utilization of the CNMF algorithm to retrieve the left ventricle blood and myocardial tissue TACs from PET images of the mouse heart. The dynamic PET image series of the LV were used to construct the input matrix *A* for CNMF, and as a result, two matrices, *W* and *H*, were calculated under the assumption that two physiological factors are mixed in each voxel. *W* is the matrix of the weights (identified to RCs and spillover fractions), and *H* contains the two physiological factors (blood and tissue factors).

As NMF returns results in arbitrary units, we conducted a normalization in two steps: (1) scaling (see Equation (5)) which ensures that the sum of the elements of *W* for each voxel is 1. In fact, this step ensures that the mixed signals sum up to the measured intensity in each voxel and, at the same time, allows the elements of *W* to be interpreted as weights. (2) Regularization parameters *α* and *β* (see Equations (1) and (2)) are chosen so that the calculated CNMF blood TAC coincides with the late blood activity. Usually, optimal values of *α* and *β* are not available without ground truth. In practice, we test a few values of these parameters and choose those that produce a CNMF blood TAC whose tail crosses the late blood sample. The model is rather insensitive to the exact values of *α* and *β* once the tail coincides with the blood sample, and no elaborate methods were needed to select appropriate values. In this study, the values used for *α* and *β* are, respectively, 1.9*E* − 05 ± 2.7*E* − 05 and 6.6*E* − 03 ± 1.4*E* − 02 (mean ± SD). As the liver is highly vascularized [54] and has relatively low [^18^F]FDG retention [12, 55], a large portion of the liver activity is contained in the blood. Consequently, the liver TAC is a reasonable approximation of the [^18^F]FDG activity in blood at the ~40 min postinjection mark. Therefore, the late portion of the liver TAC can be used as a reference of the CNMF blood factor if a blood sample is not available during measurement. These two normalization steps together ensure, to within a multiplicative constant (see Equation (6)), that the factors returned in *H* represent the tissue and blood TACs corrected for PVE and ensuing spill-in/spill-out cross-contamination, as can be clearly seen in Figure 4. Compared to CNMF, the solution obtained with UNMF (*α* = 0 and *β* = 0) is not optimal as evidenced by the TACs in Figures 4(a) and 4(d). Indeed, the extrapolation of the UNMF blood TAC tail does not coincide with the late blood sample, especially for the CTRL mouse (Figure 4(a)).

In this paper, we compared the results of CNMF with those of the RUDUR method on the 32 studied mice. This choice is motivated by the fact that the latter had demonstrated an improvement in sources separation compared to 4 other factor analysis methods [24, 37, 38] and [56]. One of them [24], in order to overcome the nonuniqueness problem, had introduced a term penalizing voxels containing a mixture of structures and as such is prone to bias in a high cross-contamination context, whereas the computed factors by the other methods are sensitive to noise outside of their proper location [36]. The comparison with RUDUR was essentially focused on the TACs (proportional to *H* rows), the weights (elements of the *W* matrix), and the values of *K*_*i*_. Regarding the TACs, the best agreement between the two methods was observed in blood TACs compared to tissue TACs especially in the AMI group (Figure 5). Although the two methods considerably reduce the peak of activity in the myocardium due to the contamination from LVC (~30–40 s postinjection), there is a clear improvement with CNMF compared to RUDUR, especially in the CTRL group (Figures 3 and 4). Another important advantage of CNMF over RUDUR is that CNMF uses only 2 regularization parameters (*α* and *β*) whereas the objective function proposed in RUDUR uses 6 [36].

Unlike the partial volume correction method based on the geometric transfer matrix [52, 57], which might be vulnerable to small errors in region of interest delineation, CNMF operates on each voxel and uses temporal information to calculate *W* and *H* matrices. The elements of *W*, for both CNMF and RUDUR, were averaged over the previously drawn ROIs on the LVC and myocardium to get recovery coefficients (*W*_*T*⟶*T*_ and *W*_*B*⟶*B*_) and spill-in fractions from one region to another (*W*_*T*⟶*B*_ and *W*_*B*⟶*T*_) (see Figure 6). It was found that infarcted mice displayed marked myocardial wall thinning and LVC dilatation [48] compared to CTRL mice. The calculated RCs *W*_*T*⟶*T*_ and *W*_*B*⟶*B*_, with both CNMF and RUDUR, are in good agreement with this structural change induced by ligation of the left coronary artery. Therefore, *W*_*T*⟶*T*_ of AMI mice is lower than that of CTRL mice, and the reverse is observed for *W*_*B*⟶*B*_. Compared to RUDUR, significant difference was observed between the two methods only in *W*_*T*⟶*T*_ and *W*_*B*⟶*T*_ for the CTRL group (Figure 6(a)). These differences are very consistent with the calculated TACs. In the CTRL group, *W*_*B*⟶*T*_ is higher in CNMF, and consequently, the remaining contamination from the LVC peak activity to the tissue TAC is reduced in CNMF compared to RUDUR. On the other hand, *W*_*T*⟶*T*_ is lower in CNMF compared to RUDUR in the CTRL group; hence, myocardial activity is more efficiently recovered in CNMF compared to RUDUR (Figures 4(b) and 4(c)).

It should be noted that the assumption that the sum of the *W* values for each voxel is 1 can be fully satisfied only if it is assumed that the signal measured in each voxel is derived exclusively from the activity in the myocardium and in the LVC. In reality, noise and surrounding tissues can contribute even marginally to the signal measured in each voxel. As a result, the constraint is expressed in Equation (5) and which should result in the following: *W*_*T*⟶*T*_ + *W*_*B*⟶*T*_ = 1 and *W*_*B*⟶*B*_ + *W*_*T*⟶*B*_ = 1 are not completely satisfied. In this study, we have found for the sum *W*_*T*⟶*T*_ + *W*_*B*⟶*T*_1.09 ± 0.02 and 0.97 ± 0.02 (mean ± SD), respectively, for the CTRL and AMI groups, and for *W_B→B_* + *W_T→B_*, we have found 0.95 ± 0.02 and 1.10 ± 0.02 (mean ± SD), respectively, for CTRL and AMI groups. These values are comparable to those found with the RUDUR algorithm. Precisely, we found for *W_T→T_* + *W_B→T_*1.06 ± 0.03 and 0.97 ± 0.02 (mean ± SD), respectively, for CTRL and AMI groups, and for *W_B→B_* + *W_T→B_*, we have found 0.95 ± 0.03 and 1.10 ± 0.02 (mean ± SD), respectively, for CTRL and AMI groups.

Reliable determination of physiological parameters, especially the [^18^F]FDG influx constant *K*_*i*_ and the metabolic rate of glucose, provides important information in drug research and development as well as medical diagnosis. We demonstrated in this study that CNMF is able to underline significant differences in *K*_*i*_ values between CTRL and AMI groups, hence highlighting metabolic changes caused by myocardial infarction. The observed increase of *K*_*i*_ on day 3 after coronary artery ligation likely indicates the presence of inflammation in the myocardium. On day 14, *K*_*i*_ becomes normal as compared to CTRL mice. Similar results were reported by Lee et al. in their study of inflammation in myocardial infarction [58]. Moreover, the calculated *K*_*i*_ values for the CTRL group using CNMF are in a good agreement with myocardial *K*_*i*_ values in literature [8, 15, 17]. No significant difference was observed between the CNMF and RUDUR *K*_*i*_ (two-tailed paired *t*-test *p* = 0.425), thus bringing more confidence in the CNMF method.

It is worth mentioning that in the current literature, it is widely believed that NMF can lead only to a local minimum because of the nonconvexity of *f*(*W*, *H*) in both *W* and *H* [59]. Nevertheless, the uniqueness of the solution still remains an open subject despite some recent attempts to address the issue [60–62]. In practice, even local minima can provide desirable properties depending on the problem.

As NMF algorithms are based on an iterative minimization process, it is well known that they are sensitive to the initialization of *W* and *H*. Currently, only very little work has been done on determining a good initialization for *W* and *H* [63], and it is standard practice to initialize NMF with random matrices. More details about NMF initialization issues can be found in [64, 65]. In this work, we initialized *H* with the scaled TACs of the ROIs drawn on the myocardium and on LVC (Figure 1 and Equation (4a)) while for *W*, we introduced the Euclidean distance between voxels and ROIs (Equation (4b)). This initialization is a reasonable starting point for both CNMF and RUDUR and ensures the stability of the solution. It must be noted that we had tested the random numbers as a starting point and, sometimes, several tests are necessary before choosing the best solution. Obviously, this will be less convenient for large data matrices.

Although the CNMF method described in this paper has been applied to cardiac [^18^F]FDG PET images of mice, it can be generalized to other tracers, organs, and species. CNMF could be especially useful for images of large animals and in humans. Despite the large size of their heart, such images are, very often, obtained from lower resolution scanners compared to the high-resolution preclinical scanners. Thus, CNMF could certainly improve the quality of image-derived time-activity curves.

Actually, not including any physiological constraint (e.g., monotonous growth of the tissue curve) may make CNMF an almost universal algorithm for separating mixed signals in voxels in a high cross-contamination context due to poor spatial resolution. However, a late blood sample is useful as a reference to compare with the CNMF solution. In the case of [^18^F]FDG, the tail of the liver TAC can be used as a more reliable surrogate for the late blood sample. In fact, the liver TAC tail is a more stable reference for adjusting the CNMF results since the blood sample is more prone to measurement errors.

One limitation of this study is the lack of validation against the arterial blood samples. Indeed, capturing the peak of the input curve under the conditions of a rapid bolus injection, as is the case in this study, presents very significant technical challenges, and our attempts to do so were unsuccessful due to mouse motion resulting from the blood sampling during the scans. Experiments with arterial blood samples in which the bolus injection is slower would be needed to compare with CNMF but at the expense of less accurate estimation of kinetic parameters.

## 7. Conclusion

In this study, we have demonstrated the ability of constrained nonnegative matrix factorization CNMF to accurately extract tissue and blood time-activity curves from [^18^F]FDG PET cardiac dynamic images of mice. Despite the small size of the mouse heart relative to the spatial resolution of PET, the CNMF algorithm was able to efficiently separate the mixed signals in image voxels. The incorporation of regularization parameters in the cost function and the scale constraint was useful in finding a solution that reflects the inherent characteristics of the desired signals and, as a result, in providing weighting factors to correct for partial volume effect and cross-contamination. Even in the mice with myocardial infarction, CNMF was able to isolate signals from different tissues. In comparison to a recently introduced approach that was advantageously validated against other competitive methods, we have observed a clear improvement of the signal separation with CNMF especially for the myocardium of normal healthy mice and also for mice with acute and chronic myocardial infarction. [^18^F]FDG influx constants were successfully assessed for the heart of both CTRL and infarcted mice using CNMF, providing realistic physiological data.

## Figures and Tables

**Table 1:** *p* values of CNMF vs. RUDUR weight comparison in the CTRL and AMI groups using one-way ANOVA with Tukey's post hoc multiple comparison test.

Group	*W*_*T*⟶*T*_	*W*_*B*⟶*B*_	*W*_*T*⟶*B*_	*W*_*B*⟶*T*_
CTRL	<0.0001	0.3948	0.1091	<0.0001
AMI	0.1359	0.9676	0.9790	0.4574

**Table 2:** *p* values of CTRL vs. AMI comparison of CNMF and RUDUR weights using one-way ANOVA with Tukey's post hoc multiple comparison test.

Method	*W*_*T*⟶*T*_	*W*_*B*⟶*B*_	*W*_*T*⟶*B*_	*W*_*B*⟶*T*_
CNMF	<0.0001	<0.0001	<0.0001	0.3672
RUDUR	<0.0001	<0.0001	<0.0001	<0.0001

## Data Availability

PET data supporting the findings of this study are available from the corresponding author upon reasonable request.

## Supplementary Materials

Supplementary Materials Table SI: CNMF and RUDUR calculated recovery factors and spillover fractions. Table SII: FDG influx constant determined using 3-compartment model applied to CNMF and RUDUR time-activity curves. Figure S1: zoom in around the one-minute time points of the time-activity curves of Figure 4.
