# Supplementary material for: Enhanced Extraction of Blood and Tissue Time-Activity Curves in Cardiac Mouse FDG PET Imaging by Means of Constrained Nonnegative Matrix Factorization
Source: Int J Biomed Imaging. 2023 Jun 15;2023:5366733. doi: 10.1155/2023/5366733 (PMC10287520; doi:10.1155/2023/5366733)
Supplement: Supplementary Materials — Table SI: CNMF and RUDUR calculated recovery factors and spillover fractions. Table SII: FDG influx constant determined using 3-compartment model applied to CNMF and RUDUR time-activity curves. Figure S1: zoom in around the one-minute time points of the time-activity curves of Figure 4. [file 5366733.f1.docx]

**Supplementary materials for:**

Enhanced Extraction of Blood and Tissue Time-activity Curves in Cardiac Mouse FDG PET Imaging by Means of Constrained Nonnegative Matrix Factorization

Otman Sarrhini^1^, Pedro D’Orléans-Juste^2^, Jacques A. Rousseau^1^, Jean-François Beaudoin^1^, and Roger Lecomte^1,3^

^1^ Sherbrooke Molecular Imaging Center, Research Center of the Sherbrooke University Hospital (CRCHUS), Sherbrooke, QC, Canada.
^2^ Department of Pharmacology-Physiology, Faculty of Medicine and Health Sciences, Université de Sherbrooke, Sherbrooke, QC, Canada.
^3^ Department of Nuclear Medicine and Radiobiology, Faculty of Medicine and Health Sciences, Université de Sherbrooke, Sherbrooke, QC, Canada.

Table SI: CNMF and RUDUR calculated recovery factors (***W_T->T_***, ***W_B->B_***) and spillover fractions (***W_T->B_***, ***W_B->T_***).

|  | **CNMF** | | | | **RUDUR** | | | |
| --- | --- | --- | --- | --- | --- | --- | --- | --- |
| **Group** | ***W_T->T_*** | ***W_B->B_*** | ***W_T->B_*** | ***W_B->T_*** | ***W_T->T_*** | ***W_B->B_*** | ***W_T->B_*** | ***W_B->T_*** |
| **CTRL** | 0.806 | 0.563 | 0.354 | 0.294 | 0.843 | 0.561 | 0.359 | 0.248 |
|  | 0.773 | 0.569 | 0.364 | 0.325 | 0.879 | 0.534 | 0.399 | 0.160 |
|  | 0.811 | 0.581 | 0.364 | 0.273 | 0.885 | 0.560 | 0.372 | 0.160 |
|  | 0.810 | 0.549 | 0.381 | 0.268 | 0.840 | 0.542 | 0.389 | 0.219 |
|  | 0.801 | 0.546 | 0.400 | 0.261 | 0.875 | 0.536 | 0.421 | 0.153 |
|  | 0.793 | 0.601 | 0.382 | 0.328 | 0.862 | 0.593 | 0.387 | 0.244 |
|  | 0.765 | 0.586 | 0.379 | 0.358 | 0.865 | 0.568 | 0.388 | 0.234 |
|  | 0.772 | 0.536 | 0.370 | 0.334 | 0.884 | 0.545 | 0.410 | 0.173 |
|  | 0.879 | 0.561 | 0.408 | 0.177 | 0.891 | 0.548 | 0.411 | 0.156 |
|  | 0.817 | 0.557 | 0.401 | 0.266 | 0.876 | 0.563 | 0.419 | 0.189 |
|  | 0.806 | 0.558 | 0.377 | 0.297 | 0.876 | 0.550 | 0.365 | 0.225 |
|  | 0.780 | 0.541 | 0.370 | 0.295 | 0.854 | 0.532 | 0.405 | 0.185 |
|  | 0.792 | 0.562 | 0.394 | 0.319 | 0.848 | 0.562 | 0.428 | 0.210 |
|  | 0.796 | 0.558 | 0.401 | 0.316 | 0.866 | 0.549 | 0.428 | 0.180 |
|  | 0.823 | 0.557 | 0.425 | 0.254 | 0.878 | 0.556 | 0.445 | 0.153 |
|  | 0.807 | 0.572 | 0.365 | 0.300 | 0.870 | 0.570 | 0.352 | 0.234 |
|  | 0.761 | 0.552 | 0.396 | 0.339 | 0.875 | 0.529 | 0.435 | 0.148 |
|  | 0.799 | 0.559 | 0.389 | 0.309 | 0.848 | 0.532 | 0.414 | 0.244 |
| **AMI3d** | 0.715 | 0.821 | 0.291 | 0.243 | 0.749 | 0.813 | 0.268 | 0.206 |
|  | 0.708 | 0.814 | 0.309 | 0.290 | 0.725 | 0.815 | 0.311 | 0.275 |
|  | 0.710 | 0.788 | 0.312 | 0.278 | 0.726 | 0.800 | 0.305 | 0.268 |
|  | 0.661 | 0.742 | 0.341 | 0.320 | 0.708 | 0.744 | 0.348 | 0.260 |
|  | 0.686 | 0.782 | 0.320 | 0.303 | 0.708 | 0.786 | 0.319 | 0.278 |
|  | 0.698 | 0.777 | 0.311 | 0.258 | 0.712 | 0.783 | 0.305 | 0.242 |
|  | 0.734 | 0.767 | 0.325 | 0.258 | 0.749 | 0.769 | 0.326 | 0.248 |
|  | 0.711 | 0.787 | 0.336 | 0.279 | 0.722 | 0.787 | 0.340 | 0.265 |
| **AMI14d** | 0.663 | 0.772 | 0.327 | 0.306 | 0.679 | 0.781 | 0.319 | 0.289 |
|  | 0.660 | 0.787 | 0.281 | 0.300 | 0.681 | 0.790 | 0.282 | 0.284 |
|  | 0.697 | 0.771 | 0.327 | 0.277 | 0.708 | 0.790 | 0.313 | 0.265 |
|  | 0.699 | 0.749 | 0.351 | 0.256 | 0.709 | 0.752 | 0.350 | 0.246 |
|  | 0.705 | 0.754 | 0.325 | 0.256 | 0.715 | 0.752 | 0.325 | 0.246 |
|  | 0.724 | 0.790 | 0.307 | 0.235 | 0.737 | 0.785 | 0.303 | 0.221 |

Table SII: FDG influx constant (*K*_i_) determined using 3-compartment model applied to CNMF and RUDUR time activity curves.

| **Group** | **CNMF *K_i_*(ml/g/min)** | **RUDUR *K_i_*(ml/g/min)** |
| --- | --- | --- |
| **CTRL** | 0.149 | 0.173 |
|  | 0.133 | 0.126 |
|  | 0.119 | 0.111 |
|  | 0.100 | 0.107 |
|  | 0.119 | 0.103 |
|  | 0.147 | 0.148 |
|  | 0.153 | 0.157 |
|  | 0.146 | 0.129 |
|  | 0.078 | 0.107 |
|  | 0.120 | 0.135 |
|  | 0.145 | 0.139 |
|  | 0.105 | 0.102 |
|  | 0.103 | 0.093 |
|  | 0.085 | 0.080 |
|  | 0.074 | 0.041 |
|  | 0.127 | 0.123 |
|  | 0.118 | 0.105 |
|  | 0.124 | 0.114 |
| **AMI3d** | 0.198 | 0.218 |
|  | 0.161 | 0.148 |
|  | 0.217 | 0.199 |
|  | 0.166 | 0.174 |
|  | 0.188 | 0.176 |
|  | 0.149 | 0.170 |
|  | 0.178 | 0.217 |
|  | 0.176 | 0.177 |
| **AMI14d** | 0.109 | 0.104 |
|  | 0.142 | 0.165 |
|  | 0.149 | 0.119 |
|  | 0.096 | 0.099 |
|  | 0.123 | 0.130 |
|  | 0.109 | 0.106 |


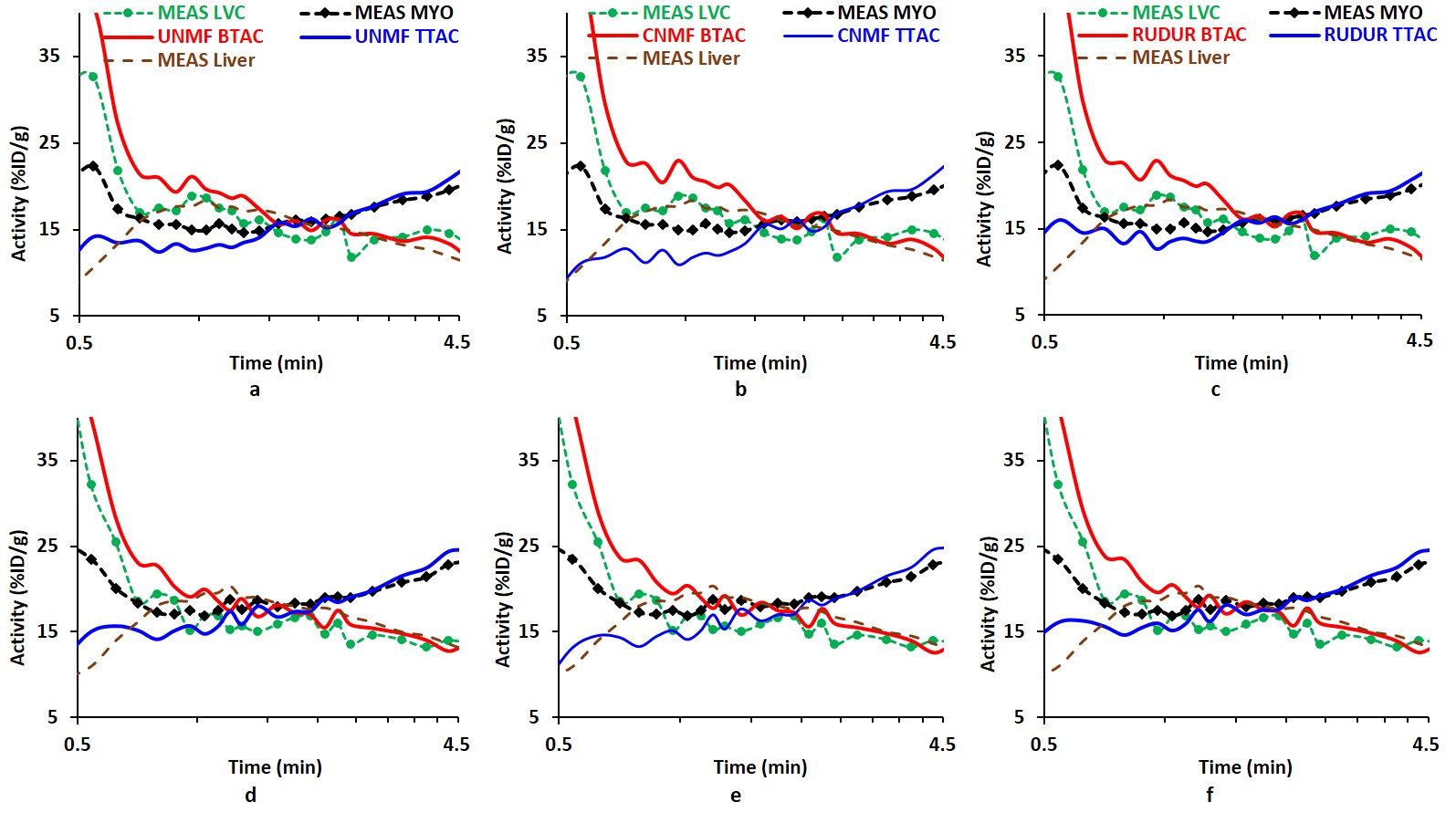


Figure S1: Zoom in around the one-minute time points of the time-activity curves of Figure 4 obtained from dynamic images of a CTRL (upper row) and an AMI3d mouse (bottom row). The measured TACs (MEAS LVC, MEAS MYO and MEAS Liver) are obtained by means of ROIs drawn on the respective organs while UNMF (a, d), CNMF (b, e) and RUDUR (c, f) TACs were obtained with their respective methods. The semi-log chart is used to better display the difference between the various curves. (*See the manuscript Figure 4 for the full scale of the axes*).
